# Counterexample Generation for Probabilistic Model Checking Micro-Scale Cyber-Physical Systems

**DOI:** 10.3390/mi12091059

**Published:** 2021-08-31

**Authors:** Yang Liu, Yan Ma, Yongsheng Yang, Tingting Zheng

**Affiliations:** 1 Institute of Logistics Science and Engineering, Shanghai Maritime University, Shanghai 201306, China; yangys@shmtu.edu.cn; 2 School of Computing, National University of Singapore, Singapore 117417, Singapore; yanma_nus@126.com (Y.M.); willow-2001@163.com (T.Z.)

**Keywords:** probabilistic model checking, micro-scale cyber-physical systems, counterexample, genetic algorithm

## Abstract

Micro-scale Cyber-Physical Systems (MCPSs) can be automatically and formally estimated by probabilistic model checking, on the level of system model MDPs (Markov Decision Processes) against desired requirements in PCTL (Probabilistic Computation Tree Logic). The counterexamples in probabilistic model checking are witnesses of requirements violation, which can provide the meaningful information for debugging, control, and synthesis of MCPSs. Solving the smallest counterexample for probabilistic model checking MDP has been proven to be an NPC (Non-deterministic Polynomial complete) problem. Although some heuristic methods are designed for this, it is usually difficult to fix the heuristic functions. In this paper, the Genetic algorithm optimized with heuristic, i.e., the heuristic Genetic algorithm, is firstly proposed to generate a counterexample for the probabilistic model checking MDP model of MCPSs. The diagnostic subgraph serves as a compact counterexample, and diagnostic paths of MDP constitute an AND/OR tree for constructing a diagnostic subgraph. Indirect path coding of the Genetic algorithm is used to extend the search range of the state space, and a heuristic crossover operator is used to generate more effective diagnostic paths. A prototype tool based on the probabilistic model checker PAT is developed, and some cases (dynamic power management and some communication protocols) are used to illustrate its feasibility and efficiency.

## 1. Introduction

**Figure 1:**
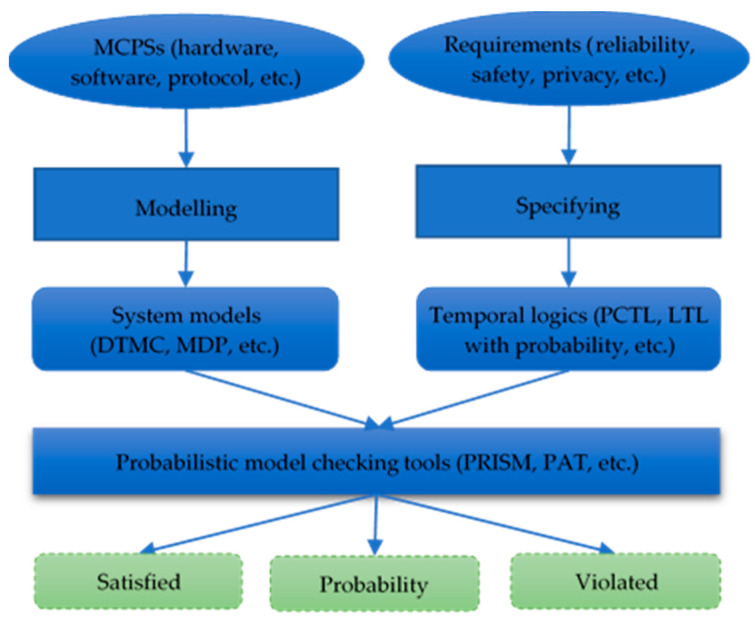
Schematic view of estimating MCPSs by probabilistic model checking.

Micro-scale Cyber-Physical Systems (MCPSs) are a special kind of CPS in micromachines, which are composed of micro/nanoscale components, and the integration of computation with physical processes in micro-/nano-assembly operations. MCPSs are about the intersections, not the union, of the physical and the cyber, and the behaviors of them are defined by both cyber and physical parts of the system [1,2,3]. Most of constituent elements of MCPSs or MCPSs themselves are usually accompanied with stochastic behaviors. The reasons for this can be classified as three aspects: (1) MCPSs contain the randomized algorithms, e.g., leader election algorithm, consensus algorithms; (2) unreliable and unpredictable system behaviors incurred by execution environment, e.g., message loss, processor failure; (3) performance evaluation by random variables assigned artificially, e.g., reliability, availability [4,5]. As an automated and complete formal verification technique at the level of system models, probabilistic model checking can be used to estimate whether the MCPSs satisfy a desired requirement property, or avoid an undesired outcome. As shown in Figure 1, MCPSs are modeled as DTMCs (Discrete-time Markov Chains), MDPs (Markov Decision Processes), PTA (Probabilistic tic Timed Automata), etc. [5]. The achieved requirement properties, e.g., function, reliability, robust, etc., are specified by PCTL (Probabilistic Computation Tree Logic), LTL (Liner Temporal logic) with probability bounds, PTCTL (Probabilistic Timed Computation Tree Logic) [5,6,7]. In this way, we can express the requirement property such as “in the MCPSs, the maximum probability to reach a set of bad states is not more than 0.001”. Some probabilistic model checking tools, such as PRISM [6] and PAT [7], have been developed and applied to quantitatively estimate, control, and synthesize the MCPSs, e.g., the mobile service robot [8], unmanned undersea vehicle [9], peacemaker [10,11,12]. As shown in Figure 1, verifying or evaluating MCPSs is a huge challenge, which involves hardware, software, communication protocols, and so on. Probabilistic model checking has the potential to address this. At present, there are two dimensions to adapt the probabilistic model checking to estimate MCPSs: (1) horizontal, extending system models, temporal logics, and corresponding algorithms to verify more complex behaviors of MCPSs; (2) vertical, optimizing the probabilistic model checking algorithm to provide more functions and better performance for verifying a certain part of MCPSs deeply.

This work belongs to the vertical dimension, which propose a counterexample generation method for probabilistic model checking MCPSs with nondeterministic and discrete-time stochastic behaviors. Up to now, existing probabilistic model checking tools cannot provide a counterexample directly. Counterexamples play a very important role in estimating or verifying MCPSs: (1) Counterexamples can feedback which parts of the system violate the requirements and provide diagnostic information; (2) Counterexamples are very effective in model-based testing, which can provide a reference for the design of test cases; (3) In the process of abstract refinement, counterexamples can provide guidance information for the refinement of rough abstract models [13,14]; (4) Counterexamples can be used to obtain the core of feasible plans in planning, such as task scheduling [15]; (5) Counterexamples are recently used to synthesize attacks for showing how confidentiality of systems can be broken, and the quality assurance of multi-agent systems [16].

### 1.1. Related Works

The counterexample in probabilistic model checking may be a set of diagnostic paths that satisfy a given property, and the cumulative probability mass does not satisfy a given bound. It cannot be generated during the process of probabilistic model checking and needs the dedicated algorithm [17]. We divide the existing works into two kinds of methods for generating a counterexample in probabilistic model checking: the accurate and approximate approach.

#### 1.1.1. Accurate Approach

Han, Katoen, et al. [18] provide the theoretical and algorithmic foundations for counterexample generation in probabilistic model checking, which is the earliest research on the counterexample in probabilistic model checking. They define the counterexample for DTMC and translate the counterexample solving problem into the shortest path problem in the corresponding directed weight graph. Thus, the counterexample generation can explicitly enumerate the paths according to the probability they carry. To obtain the smallest counterexample, the enumeration stops when the cumulative probability of all generated paths exceeds the probability bound set in advance. Algorithmically, this can be done efficiently by translating this problem into a *k*-Shortest-Paths problem, where *k* is not fixed in advanced and is determined during the calculation. Eppstein and REA algorithms are applied to generate a counterexample for DTMC. On the basis of [19], the approach of calculating and representing a counterexample in a succinct way using a regular expression is presented in [20]. According to [20], the regular expressions that represent a counterexample can be calculated by the state elimination method of the automaton theory, which is guided by the *k* shortest paths search. The counterexample can also be compacted based on the *strongly connected components* (SCCs), and the obtained acyclic model is helpful in reducing efforts to determine the counterexample. 

A hierarchical counterexample generated by performing SCC reduction is presented in [21]. Considering the unbounded-until property formula $P_{\leq p}\left(\Phi U^{\leq h}\Psi \right)$, where h=∞, counterexample generation can be carried out by applying *k* shortest paths algorithms such as Epstein algorithm, and the time complexity of which is O(a+blogb+c), where *a* is the number of states and *b* is the number of transitions of DTMC. For bound-until property $P_{\leq p}\left(\Phi U^{\leq h}\Psi \right)$, where h∈N, a recursive enumeration algorithm to generate counterexample is presented in [20]. Reference [22] defines the counterexample of MDP as the general DTMC. Lal and Prabhakar [14] use a counterexample generated by this method to guide the abstraction-refinement for the polyhedral probabilistic hybrid systems.

Taking PCTL path formula $\Phi_{1}U\Phi_{2}$ as an example, the process of generating a counterexample for MDP by the Eppstein algorithm can be summarized as follows: (1) make $\Phi_{2}$-states and all $\neg \Phi_{1}\wedge \neg \Phi_{2}$-states absorbing, (2) insert a sink state and redirect all outgoing edges of $\Phi_{2}$-states to it, (3) turn it into a weighted digraph, (4) implicit representation of paths, (5) represent paths by a heap, (6) find the k shortest paths as the counterexample.

#### 1.1.2. Approximate Approach

A critical subsystem, as a subsystem of DTMC, can represent a counterexample for probabilistic model checking. It is called a minimal critical subsystem if the number of states and transitions included is minimal compared to other subsystems, and the smallest subsystem if it is minimal and carries a maximal probability to reach a target state. Chadha et al. [23] further define the counterexample as the general MDP and give the corresponding algorithm for solving the minimal counterexample. This is the first work of counterexample generation for MDP. 

Generating the smallest critical subsystems to represent the counterexample is an NP-complete problem which has been proved in [23,24], and it is hard to solve with exact and complete algorithms. A heuristic search method like best first search to obtain a counterexample is presented in [25,26]. However, it may not be a smallest, or even a minimal counterexample. Symbolic methods like bounded model checking (BMC) for finding a small critical subsystem have been developed in [25,26]; it uses a symbolic representation to reduce the size of the counterexample. Another option to determine the smallest critical subsystem is to use mixed integer linear programming techniques in [27,28]. Aljzzar et al. [29] propose a directed state space search method called XBF (XZ, XUZ) to generate a counterexample, which is the first work of counterexample generation with a heuristic. There are some works in this direction, such as [13,30,31], which optimizes the heuristics to generate a counterexample for DTMC.

XBF extends the search strategy BF (Best-First) to generate a counterexample for MDP. In the framework of generating a counterexample for MDP by the Eppstein algorithm, XBF makes the following three modifications: (1) XBF records all parent states for each state; (2) XBF maintains a graph which contains, at any point in the search, the currently selected diagnostic subgraph; (3) when XBF finds the first target state, it continues the search for further target states until the whole state space has been processed, or termination is explicitly requested.

### 1.2. Our Contribution

This paper deals with counterexample generation for probabilistic model checking MCPSs with nondeterministic and discrete-time stochastic behaviors, in which MCPSs are modeled as MDP, and the requirements are specified as PCTL. Solving the smallest counterexample for probabilistic model checking MDP has been proven to be an NPC problem. Although some heuristic methods are designed to generate the counterexample for MDP, it is usually difficult to fix the global heuristic functions. Instead, we design the local heuristic function integrated into the Genetic algorithm (heuristic Genetic algorithm, HGA) to explore the state space, and propose a counterexample generation method for probabilistic model checking MDP against requirement property in PCTL. We use a diagnostic subgraph to represent the compact counterexample for MDP, and exploit diagnostic paths to constitute an AND/OR tree for constructing the diagnostic subgraph. We adopt the indirect path coding of the Genetic algorithm to extend the search range of state space, and the heuristic crossover operator to generate more effective diagnostic paths. A corresponding prototype tool is implemented based on PAT [16], and some MCPSs cases are used to illustrate its feasibility and efficiency.

### 1.3. Outline of the Paper

The rest of this paper is organized as follows. In Section 2, we introduce some preliminaries and definitions. Section 3 describes how to optimize the Genetic algorithm with a heuristic to generate a counterexample for MCPSs model MDPs. In Section 4, we show an experimental evaluation of some MCPSs cases, in order to illustrate the feasibility and efficiency. Conclusion and future work are in Section 5. 

## 2. Preliminaries

### 2.1. MDP

An MDP can be viewed as an extension of DTMC, which permits both probabilistic and non-deterministic choices. In an MDP, each transition includes a non-deterministic choice of actions in state *s*. Formally, an MDP is defined as follows.

**Definition** **1** (MDP). *Let AP be a set of atomic propositions. A Markov decision process M is a tuple* $\left(S,s_{init},A,P,L\right)$*, where S is a finite set of states,* $s_{init}\in S$ *is the initial state, A is a set of actions,* P: S×A×S→[0, 1] *is a probability transition function such that for every state,* s∈S *and an action* α∈A: $\sum s^{\prime }\in S,\;\;P\left(s,\alpha ,s\prime \right)\in \left\{0,\;1\right\}$*, and* $L:S\to 2^{AP}$ *is a labeling function of atomic propositions.*

For a state s∈S, the probability of *s* to its successor state s′ by action *a* is given by P(s,α,s′). If and only if $\sum_{s^{\prime }\in S}P\left(s,\alpha ,s\prime \right)=1$, we call the action α enabled in the state *s*, otherwise, the action α is disabled. We use A(s) to represent a set of actions that are enabled at state *s.*

A path represents the execution of the system modeled by MDP; that is, the possible event behavior of the system, which can be described as an infinite sequence of states.

**Definition** **2** (Path). *An (infinite) path of MDP M is an infinite sequence* $\sigma =s_{0}\overset{\alpha_{0}}{\to }s_{1}\overset{\alpha_{1}}{\to }s_{2}\ldots$ *with* $\alpha_{i}\in A\left(s_{i}\right)$ *such that* $P\left(s_{i},\alpha_{i},s_{i+1}\right)>0$ *for all* i≥0.

We use len(σ) to denote the length of σ, which is determined by the number of states. For an infinite path σ, len(σ) is ∞. For a natural number *i* such that 0≤i<len(σ), σ[i] refers to the *i*th state of σ, i.e., $s_{i}$. Using $\sigma^{\left(i\right)}$ to indicate the *i*th prefix of σ, formally, $\sigma^{\left(i\right)}=s_{0}\overset{\alpha_{0}}{\to }\ldots \overset{\alpha_{i-1}}{\to }s_{i}$, which represents the *i* + 1 state of the path σ. For 0≤i≤len(σ), $A_{\sigma }\left(i\right)$ denotes the *i*th action in σ, namely $\alpha_{i}$. For a finite path, last(σ) denotes the last state of σ. $Paths^{M}$ and $Paths_{fin}^{M}$ represent the set of infinite paths and the set of all finite paths in *M*, respectively. $Paths^{M}\left(s\right)$ denotes the set of infinite paths which start at s and $Paths_{fin}^{M}\left(s\right)$ denotes the set of finite paths starting from *s*. Non-determinism in an MDP is resolved by schedulers (also called adversary, policy, or strategy). A scheduler for an MDP $M=\left(S,s_{init},A,P,L\right)$ is a function mapping every finite path σ in *M* onto an action d(σ)∈A(last(σ)). According to [32], we consider the deterministic scheduler that can deterministically select actions, which induces the maximal and minimal probability measures. The scheduler converts MDP into DTMC for which the probability of paths is measurable. We refer to the set of infinite paths under this schedule as $Paths_{d}\left(s_{0}\right)$. Paths in an MDP are called valid if the paths are allowed under a given scheduler *d*. We give the definition of the valid path as follows.

**Definition** **3** (Valid path). *A finite or an infinite path* σ *in an MDP is valid under a scheduler* d*, if and only if for all i,* 0≤i≤l(σ)−1*, it holds that* $A_{\sigma }\left(i\right)=d\left(\sigma^{\left(i\right)}\right)$ *and* $A_{\sigma }\left(i\right)\left(s_{i+1}\right)>0$*. Otherwise, the path* σ *is invalid under scheduler d.*

The underlying σ-algebra is formed by the cylinder sets which are induced by finite paths under the scheduler denoted $FinitePaths_{d}\left(s_{0}\right)$. The probability of this cylinder set is computed by using the following equation:

(1) $${Pr}_{d}(\sigma \in FinitePaths_{d}\left(s_{0}\right)|\sigma =s_{0}\overset{\alpha_{0}}{\to }\ldots \overset{\alpha_{i-1}}{\to }s_{i})=\Pi_{0\leq i\leq n}P\left(s_{i},\alpha_{i},s_{i+1}\right)$$

**Figure 2:**
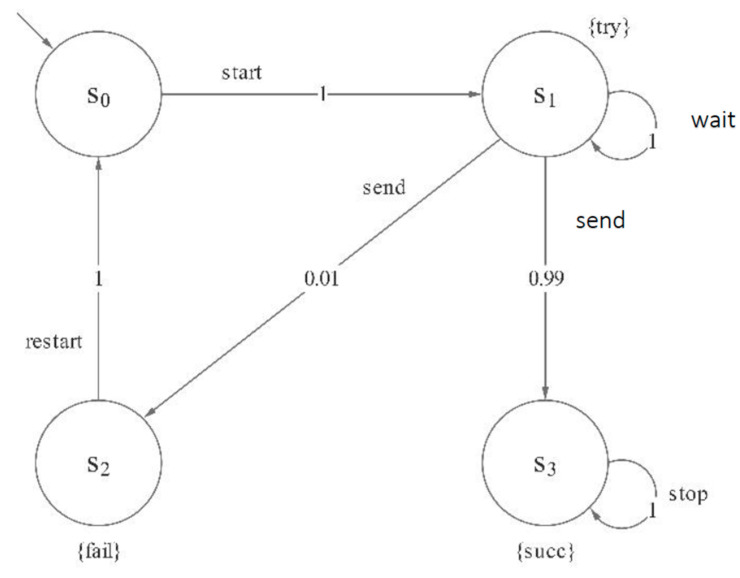
MDP model of a simple communication protocol.

**Example** **1.** *Figure 2 is an illustrative example for MDP, which models a simple communication protocol in MCPSs. In this MDP, state set*$S=\{s_{0}$, $s_{1}$, $s_{2}$, $s_{3}\}$*, A* = {start, wait, send, restart, stop}*, atomic propositions set AP* = {try, succ, fail}*, L(*$s_{0}$*) =* ∅*, L*($s_{1}$) = {try}, *L*($s_{2}$) = {fail}, *L*($s_{3}$) = {succ}. $s_{0}$ *is the initial state; it starts trying to send a message after one step. Then, there is a nondeterministic choice between: (1) waiting a step as the channel is busy, i.e., path* $\sigma =s_{0}\overset{start}{\to }s_{1}\overset{wait}{\to }s_{1}$*, (2) sending the message. If the latter, it is to send successfully with probability 0.99 and stop, i.e., path* $\sigma =s_{0}\overset{start}{\to }s_{1}\overset{send}{\to }s_{3}\overset{stop}{\to }s_{3}$*; and it is failed to send with probability 0.01 and restart, i.e., path* $\sigma =s_{0}\overset{start}{\to }s_{1}\overset{send}{\to }s_{2}\overset{restart}{\to }s_{0}$.

### 2.2. Probabilistic Computation Tree Logic

We consider the calculation of the (constrained) reachability probability in MDP. Let a finite MDP $M=\left(S,s_{init},A,P,L\right)$ and B⊆S a set of a target. The maximal probability of reaching a state in *B* from the initial state of MDP is equivalent to determining ${Pr}^{max}\left(s{⊨}_{M}◊B\right)=sup_{D}Pr\left(s⊨◊B\right)$. Note that the supremum ranges over all, potentially infinitely many, schedulers for *M*. Probabilistic computation tree logic (PCTL) is a probabilistic branching-time temporal logic. PCTL state formulae over the atomic propositions set *AP* are formed according to the following grammar:

$$\Phi ::=true\;\left|\;a\;\right|\;\Phi_{1}\wedge \Phi_{2}\;\left|\;\neg \Phi \;\right|\;P_{\sim p}\left(\phi \right)$$

where a∈AP, ϕ is a path formula, ~∈{<,≤,>,≥}, and p∈[0,1]. PCTL path formula is formed according to the following grammar:

$$\phi ::=\Phi_{1}U\Phi_{2}\;|\;\Phi_{1}U^{\leq n}\Phi_{2}$$

where $\Phi_{1}$ and $\Phi_{2}$ are state formulae, and $n\in {\mathrm{IN}}_{\geq 0}U\left\{\infty \right\}$. The formula $\Phi_{1}U\Phi_{2}$ means that $\Phi_{2}$ is satisfied and all preceding states satisfy $\Phi_{1}$. The path formula $\Phi_{1}U^{\leq n}\Phi_{2}$ is the step-bounded variant of $\Phi_{1}U\Phi_{2}$. n=∞ in a path formula means that it is unbounded until formula. 

Let s∈S be a state and σ be an infinite path under *D*, where *D* denotes the set of all schedulers in MDP; the semantics of PCTL formulas over MDP are defined by satisfaction relation ${⊨}_{D}$:

$$s{⊨}_{D}P_{\sim p}\left(\phi \right)\;\mathrm{iff}\;{Pr}_{s}^{D}(\{\sigma \in Path^{D}\left(s\right)|\sigma {⊨}_{D}\phi \})\sim p$$

$\sigma {⊨}_{D}\Phi U^{\leq h}\Psi$ iff ∃i≤h, such that $\sigma \left[i\right]{⊨}_{D}\Psi$ and $\exists \forall j<i:\sigma \left[j\right]{⊨}_{D}\Phi$.

We abbreviate ${Pr}_{s}^{D}(\{\sigma \in Path^{D}\left(s\right)|\sigma {⊨}_{D}\phi \})$ as ${Pr}_{s}^{D}(\phi )$ for convenience. It needs to calculate the probability of each path under *D* starting from s and satisfying ϕ, and determine which state satisfies the formula $P_{\sim p}\left(\phi \right)$. We use the set $Sat\left(P_{\sim p}\left(\phi \right)\right)$ to represent all states that satisfy $P_{\sim p}\left(\phi \right)$. For MDP $M=\left(S,s_{init},A,P,L\right)$, a formula $P_{\sim p}\left(\phi \right)$ is satisfied if and only if for every $d\in D:{Pr}_{d}(\phi )\sim p$, where ${Pr}_{d}(\phi )$ denotes the probability of the set of all finite paths satisfying ϕ under scheduler *d*. The probability of paths in MDP is only defined in a given scheduler *d*. Probabilistic model checking MDP should consider the maximizing or minimizing probability values incurred by the different schedulers. Let ${Pr}^{max}\left(\phi \right)$ denote the maximal probability mass at which a MDP *M* satisfies ϕ, ${Pr}^{max}\left(\phi \right)=max\left({Pr}_{d}(\phi \right)|d\in D)$, and dually, the minimal probability ${Pr}^{min}\left(\phi \right)=min\left({Pr}_{d}(\phi \right)|d\in D)$. For properties in the upper bound, it is obvious that $M⊨P_{\leq p}\left(\phi \right)\Leftrightarrow {Pr}^{max}\left(\phi \right)>p$.

### 2.3. Genetic Algorithm

The Genetic algorithm is a heuristic search, inspired by Charles Darwin’s theory of natural evolution. This algorithm reflects the process of natural selection where the fittest individuals are selected for reproduction in order to produce offspring of the next generation. The Genetic algorithm has been applied to a board range of learning and optimization problems [33]. It begins with a set of a random population of coded candidate solutions which are called the chromosome. Then, the fitness is evaluated, measured by the fitness function of each candidate solution in the current population, and the fittest candidate solution is selected as parent of the next generation. The next step is to generate a second-generation population of solutions from those selected through crossover and mutation. The fittest parents and the new offspring form a new population. We give the standard Genetic algorithm in pseudocode, as shown in Algorithm 1.
**Algorithm 1.** Standard Genetic algorithm.STARTGenerate the initial populationCompute fitnessREPEAT       Selection       Crossover       Mutation       Compute fitnessUNTIL population has convergedSTOP

## 3. Counterexample Generation with Heuristic Genetic Algorithm

In this section, we adopt a diagnostic subgraph to represent the counterexample, and optimize the Genetic algorithm with heuristic (heuristic Genetic algorithm, HGA) to generate a counterexample for probabilistic model checking MCPSs. The system model of MCPSs is MDP, and the requirement property is expressed by PCTL, i.e., $P_{\sim p}\left(\phi \right)$. We will focus on the formula $P_{\leq p}\left(\phi \right)$ in PCTL; the lower bounded formulas $P_{>p}\left(\phi \right)$ and $P_{\geq p}\left(\phi \right)$ can be converted to upper bound formulas.

### 3.1. Counterexample Represented by Diagnostic Subgraph

In classical model checking, the checked property will determine the shape of a counterexample. For linear temporal logic such as LTL, a failure path serves as a counterexample; and for CTL, the path as a counterexample is only for a subclass of global quantifier formulas. In probabilistic model checking, the situation is very complex. Let $\phi =\Phi_{1}U^{\leq n}\Phi_{2}$, $s{⊭}_{D}P_{\leq p}\left(\phi \right)$, if and only if$\;{Pr}_{s}^{D}(\phi )>p$ holds. Consequently, if $P_{\leq p}\left(\phi \right)$ does not satisfy at state *s,* then the probability sum of all paths which satisfy formula ϕ with initial state *s* exceeds the probability bound *p*. It is obvious that the counterexample can be represented by finite paths. For a low-bounded property formula $P_{\geq p}\left(\phi \right)$, the definition of the counterexample does not make sense, since the empty set satisfies this condition; diagnostic path ${Pr}_{s}^{D}(\phi )<P$ does not carry useful information. Therefore, in the following, we only consider the upper bound. 

The PCTL property $P_{\leq p}\left(\phi \right)$ is refused in an MDP, if there exists at least one scheduler *d* such that the probability mass of the paths $FinitePaths_{d}\left(s_{0}\right)$ satisfying ϕ under *d* exceeds *p*, where $FinitePaths_{d}\left(s_{0}\right)$ means a set of paths starting from state $s_{0}$ and satisfying ϕ under a scheduler *d.* We denote this set by $FinitePaths_{d}\left(s_{0}⊨\varphi \right)$. These finite paths are also called diagnostic path.

**Definition** **4** (Diagnostic paths). *Let M be an MDP model of a MCPS, and upper bounded formula* $P_{\leq p}\left(\phi \right)$ *be a requirement property under consideration. A counterexample of the property* $P_{\leq p}\left(\phi \right)$ *for MDP M is a set X of diagnostic paths in M such that* ${Pr}^{max}\left(\phi \right)>p$ *holds.*

**Figure 3:**
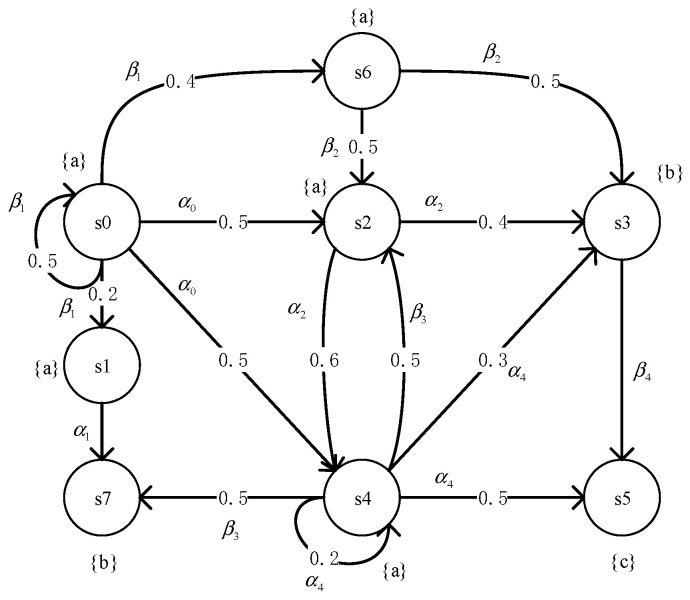
A Markov decision process.

**Example** **2.** *Let us consider the example of MDP shown in Figure 3 and the property*$P_{\leq 0.4}\left(aUb\right)$. *This property is violated in this model since there exists a scheduler that induces a set of diagnostic paths that satisfy formula* aUb*and their probability mass greater than 0.4*. *We have the following diagnostic paths:*$\sigma_{1}=s_{0}\overset{\alpha_{0}}{\to }s_{2}\overset{\alpha_{2}}{\to }s_{3}$, $\sigma_{2}=s_{0}\overset{\alpha_{0}}{\to }s_{4}\overset{\alpha_{4}}{\to }s_{3}$, $\sigma_{3}=s_{0}\overset{\alpha_{0}}{\to }s_{2}\overset{\alpha_{2}}{\to }s_{4}\overset{\alpha_{4}}{\to }s_{3}$*and their respective probabilities are 0.2, 0.15, 0.09. The set of three diagnostic paths is* $C=\left\{\sigma_{1}\sigma_{2}\sigma_{3}\right\}$*, and the probability is 0.44, which is greater than the given probability bound of 0.4. Thus, set C is a counterexample of the property of model violation.*

We argue that a counterexample in probabilistic model checking should satisfy the following three conditions: (1) explain why the probabilistic system does not satisfy the property; (2) represent violation of a class of requirement properties; (3) identify errors in the system simply and specifically. A set X of diagnostic paths can show the violation of a property, but it contains too much redundancy information. Thus, we define the diagnostic subgraph to represent a counterexample for MDP. 

An MDP can be regarded as a weighted directed graph where the vertices of graph represent states of MDP and the edges of graph represent transitions of MDP. Thus, the transition probability between states in the MDP can be converted into the weight between the vertices in the graph.

**Definition** **5** (Subgraph of MDP). *Let* G=(V,E,W) *be a weighted digraph generated by an MDP* $M=\left(S,s_{init},A,P,L\right)$*; a subgraph of M is* $G\prime =\left(V^{\prime },E^{\prime },W\prime \right)$*, where G’ is a part of G with* $V^{\prime }\subseteq V,\;E^{\prime }\subseteq E,\;W\prime \subseteq W$ *such that* $\left\{\left(s,s\prime \right)\subseteq E^{\prime }|\forall s,s^{\prime }\subseteq V^{\prime }\;,\;\left(s,s^{\prime }\right)\subseteq E\right\}$.

The subgraph of MDP not only contains states and transitions of paths, but also all transitions connecting them in the original MDP.

**Definition** **6** (Diagnostic subgraph). *Let* $M=\left(S,s_{init},A,P,L\right)$ *be an MDP; the counterexample for* $s{⊭}_{D}P_{\leq p}\left(\phi \right)$ ($s{⊭}_{D}P_{<p}\left(\phi \right)$) *is a diagnostic subgraph such that* $Pr_{s}^{D}\left(\phi \right)>p$ ($s{⊭}_{D}P_{\geq p}\left(\phi \right)$) *does not hold.*

**Theorem** **1.** *For MDPs, a Counterexample represented by the diagnostic sub-graph, has less than or equal to the state space of the counterexample represented by diagnostic paths.*

**Proof** **of** **Theorem** **1.** If a counterexample for MDP is one diagnostic path, it also can be represented with a diagnostic subgraph with this diagnostic path. If a counterexample for MDP is a measurable subset$\;X=\left\{X_{1},X_{2}\ldots X_{n},\right\}\subseteq \{\pi \in FinitePaths_{d}\left(s\right)|\pi {⊨}_{D}\phi \}$ such that$\;Pr_{s}^{D}\left(\phi \right)>p$ ($s{⊭}_{D}P_{\geq p}\left(\phi \right)$), each path in the measurable subset can be derived from a diagnostic loop path. We can get that the probability of a diagnostic loop path is greater than or equal to the diagnostic path. Thus, the state space of the counterexample represented by a diagnostic subgraph is less than or equal to the counterexample represented by the diagnostic paths. □

### 3.2. Genetic Algorithm with Heuristic (HGA)

The most critical issue of counterexample generation with HGA is how to code paths in the weighted directed graph for MDP as a chromosome. In order to represent all possible paths in the graph, we adopt a priority-based coding method. Then, we use heuristic crossover operators to generate better individuals than the parents, which can improve the search speed. We calculate the shortest path by the fitness function. 

#### 3.2.1. Coding the Path

According to [34,35], there are two main coding schemas of the Genetic algorithm for solving the shortest path, namely, direct and indirect coding. Direct coding is based on the identification number of the node. The disadvantage of this coding is that an invalid path is generated because the random sequence of node identification numbers may not correspond to a valid path. Therefore, direct coding is not a good choice. We choose priority-based indirect coding, which is used in [35,36].

**Figure 4:**
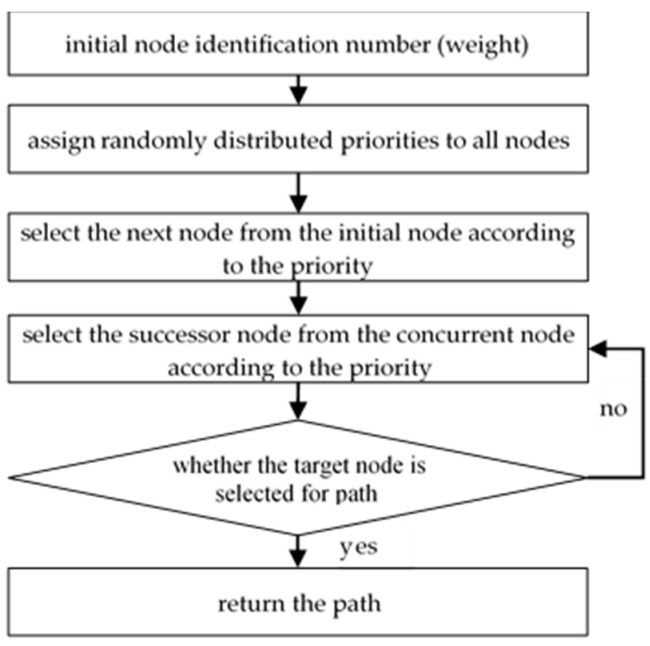
Indirect coding flow.

Indirect coding has significant advantages in generating finite paths compared to direct coding schemes. The indirect coding scheme used in the Genetic algorithm is shown in Figure 4. The initial node identification number appears directly on the path; it is different from the path that uses the instruction information about forming the path node. The guidance information is the priority of each node in a weighted directed graph. In the initialization phase, the priorities are randomly distributed. The path starting from the initial node and terminating at the target node is generated by a sequential node attach procedure. In each step of the path construction, if there are several successor nodes to consider, then the node with the highest path priority is selected. Repeat the above process until reaching the target node.

#### 3.2.2. Fitness Function

The path quality is measured by the fitness function. In order to make the counterexample as small as possible, the goal of the fitness function is to minimize the search for the candidate path. The fitness function is described as follows:

(2) $$f={\left(\underset{e\in C}{\sum }\omega \left(e\right)\right)}^{-1}$$

where *C* is the path set in the graph, that is, each chromosome in the Genetic algorithm. e is an edge of path *C*, and ω(e) is the weight of the edge.

The construction process of diagnostic paths is selecting individuals with higher fitness from the current population and eliminating individuals with lower fitness. Assuming that the population size is *N* and fitness of the *i*th chromosome is $p_{i}$, then the probability of which the *i*th chromosome is selected is

(3) $$p_{i}=\frac{f_{i}}{\sum_{i=1}^{N}f_{i}}$$

#### 3.2.3. Heuristic Crossover Operator

The crossover operation replaces the reorganization of the partial structure of two parent individuals to generate a new individual. This paper uses a heuristic crossover approach. For the cross of individuals $c_{1}$ and $c_{2}$, we firstly generate a cross break-point *K* randomly, then copy the *K*th to *N*th gene string $c_{11}$ in $c_{1}$ to the back of $c_{2}$ and delete the same gene in $c_{2}$ as in $c_{11}$; the same process is performed to $c_{2}$, the *K*th to *N*th gene string $c_{22}$ of $c_{2}$ is copied to the back of $c_{1}$, and the same gene in $c_{11}$ as in gene string $c_{22}$ is deleted. This produces two legal intermediate individuals $c_{3}$, $c_{4}$ then selects two individuals with high fitness as crossover offspring from $c_{1}$,$\;c_{2}$,$\;c_{3}$, $c_{4}$ and puts the selected descendants into the mating pool for the next crossover.

#### 3.2.4. Mutation Operator

The mutation operation is to change the gene values of certain gene spots on chromosome individuals in the population. Its purpose is to enable Genetic algorithms to have local random search capabilities and maintain group diversity. Suppose we perform mutation operations on individual *A*: [$s\;a_{1}\;a_{2}\;a_{3}\;a_{4}\;a_{5}\;t$]; select a gene block *Y:* [$a_{2\;}a_{3}\;a_{4}\;a_{5}$] on *A* first, and then randomly generate a path *X*: [$a_{2\;}R\;a_{5}$] from $a_{2}$ to $a_{5}$ in graph, where *R* represents all genes on the path *X*, i.e., diagnostic paths. Then, individual *A* after the mutation operation is [$s\;a_{1}\;a_{2}\;R\;a_{5}\;t$].

### 3.3. Generating Counterexample with HGA

The diagnostic paths are added in the set *R*, and we need to filter out the invalid paths in *R*. We call the set of all diagnostic paths from *R* which are valid under *d* the maximum of *R.* The goal is to provide a counterexample that contains only valid paths.

**Definition** **7** (Maximum of R). *Let d be a maximizing scheduler of R, and the set of all diagnostic paths which are valid under d is called a maximum of R*.

If *R* is a counterexample, then each maximum of *R* only contains the valid paths. Now, we need to compute a maximum *X* of R and to compute its probability ${Pr}^{max}\left(X\right)$. To this end, we define the compatible paths as follows.

**Definition** **8** (Compatible paths set). *A set of paths C is called compatible, if and only if there exists a scheduler d such that all paths in C are valid under d*.

We proposed this concept because each scheduler compatible subset *X* of *R* with maximal probability is a maximum of *R*. For diagnostic paths, the first prefix after the common prefix is decisive for scheduler compatibility. When a branch exists in a state, the diagnostic path is not compatible. We can always define a scheduler that allows a set of diagnostic paths to branch in an action. Therefore, the diagnostic path of the branch in the action is compatible. Checking the scheduler compatibility can be done as follows. *R* can be implemented as an AND/OR tree in order to check scheduler compatibility. Each diagnostic path is stored in *R* by mapping its states and actions to nodes. The OR nodes map to the state nodes, in which the scheduler makes a decision. The AND nodes map to the probability decisions after an action has been chosen by the scheduler. For a searched diagnostic path $\sigma =s_{0}\overset{\alpha_{0}}{\to }\ldots \overset{\alpha_{k-1}}{\to }s_{k}$, we interpret *σ* as an alternating sequence of OR and AND nodes, i.e., $S_{\sigma }=\left(s_{0},\alpha_{0}\ldots \alpha_{k-1},s_{k}\right)$. If *R* is empty, we add all nodes and edges in diagnostic path σ directly to *R*. If *R* is not empty, determine whether the longest prefix of $S_{\sigma }$ is already included in *R*, which starts from root $s_{0}$, i.e., whether the longest prefix is shared by any path in $S_{\sigma }$ and *R*. When the longest prefix is included in the tree, insert diagnostic path σ into the tree. The remainder of diagnostic path σ becomes a new subtree, with the node ending with the longest prefix as root. Then, assign a probability to each node of this diagnostic path σ in a bottom-up manner. For AND root node in diagnostic path σ, its probability value is the sum of the values of its child nodes. The value marked on the leaf is the probability of the path from the root to it. The value marked in the internal AND node is the sum of values of its child nodes, and the value of the OR node is the maximum value of all child node values. Algorithm 2 illustrates the flows to a counterexample generation for MDP with HGA.
**Algorithm 2.** Counterexample generation for MDP with HGA.Step1: Initialize *R* and *X* as an empty set, respectively, that is, without edges and nodes.Step2: Generate a diagnostic path σ by HGA in Section 3.2Step3: Replace diagnostic path σ with the sequence $S_{\sigma }=<s_{0},\alpha_{0}\ldots s_{i}\alpha_{i}>$ in the AND/OR treeStep4: If *R* is empty, then directly add the nodes and edges in the σ and go to step 8.Step5: Determine whether the longest prefix in $S_{\sigma }\;$already exists in *R*, starting from the initial node $s_{0}$.Step6: Add the remainder of $S_{\sigma }\;$to the last node in the longest prefix.Step7: Assign mark $M_{p}$ to each node in *R* from the bottom up, where $M_{p}$ is the probability of each nodeStep8: If a node n in $S_{\sigma }$ is an AND node, then its probability value $M_{p}$ is the sum of the child-nodes’ value.Step9: If a node n in $S_{\sigma }$ is an OR node, then its probability value $M_{p}$ is the maximum of the child-nodes’ value.Step10: If ${Pr}_{max}(X)>p$ holds, return the counterexample.Step11: Go to Step2

**Theorem** **2.** *The Algorithm 2 will terminate after generating a counterexample*.

**Proof** **of** **Theorem** **2.** Assume that Algorithm 2 does not terminate, only an infinite counterexample for $s|\neq_{D}P_{\leq p}\left(\phi \right)$ is generated, i.e., the counterexample consists of an infinite path. Let $C=\left\{\sigma_{1},\sigma_{2}\ldots \right\}$ be an infinite counterexample; we have $\sum_{i=1}^{\infty }Pr\left(\sigma_{i}\right)=\underset{j\to \infty }{lim}\sum_{i=1}^{j}Pr\left(\sigma_{i}\right)>p$, where $\sum_{i=1}^{\infty }Pr\left(\sigma_{i}\right)=L$ and $\sum_{i=1}^{j}Pr\left(\sigma_{i}\right)=a_{j}$. According to the characteristics of the limit, this means that:

(4) $$\forall \varepsilon >0.\;\exists N\in \mathrm{IN}.\;\forall n\geq N.\;\left|a_{n}-L\right|<\varepsilon$$

Let 0<ε<L−p. According to (3), for some n≥N, $\left|a_{n}-L\right|<\varepsilon \Rightarrow |a_{n}-L|<L-p$; but the finite set $C\prime =\left\{\sigma_{1},\sigma_{2}\ldots \sigma_{n}\right\}$ is also a counterexample as Pr(C′)>p. This is a contradiction. Therefore, the algorithm will terminate after generating a counterexample. □

### 3.4. An Example

**Figure 5:**
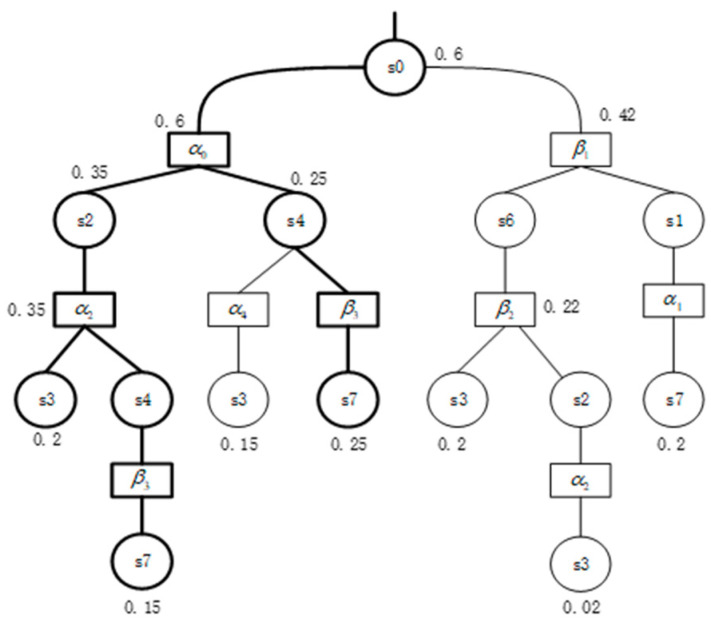
The AND/OR tree.

We consider the MDP as shown in Figure 3 and the property $P_{\leq 0.5}\left(aUb\right)$. The AND/OR tree of it is shown in Figure 5. Searching the state space by HGA and generating the most probable diagnostic paths, then add it to the AND/OR tree, which is the implementation of *R.* $s_{2}$ is the AND node, the probability of which is the sum of its child-nodes’ value, i.e., 0.35. $s_{4}$ is the OR node, the probability of which is the maximum of child-nodes’ value, i.e., 0.25. The diagnostic paths we are interested in are marked with thick lines. The number identified next to each node is based on the probability calculated in Algorithm 2, the probability of the root node is 0.6, which means the maximum probability of identification is 0.6. Since 0.6 > 0.5, the maximum that was obtained is a counterexample, as shown in Figure 5.

## 4. Experimentation

We develop a prototype tool CX-HGA for counterexample generation with HGA, based on PAT [7,16]. It is developed in the Java language with explicit-state data structures (sparse matrices, bit-sets, etc.). MDPs are described with PAT language, and the probability of the diagnostic subgraph is calculated by the PAT engine. All experiments are performed on a machine with Intel Pentium CPU 3.2 GHz speed and 1 GB memory. In our algorithms, population size is set to 100 and the evolution generation is 3000. Crossover probability is 0.90 and mutation probability is 0.10. By some PRISM Benchmark cases, we compare HGA with the XBF-based (XZ and XUZ) algorithm and Eppstein algorithm (Eppstein) for counterexample generation, which are implemented in DIPRO [37]. We also compare HGA and Genetic algorithm (GA) with directed coding. Note that DTMCs are a proper subset of MDPs. Any Markov chain is an MDP in which, for any state *s*, *A* (*s*) is just a singleton set. Thus, the experiments also include two DTMC cases for comparing with the existing works. We select 4 cases for demonstrating the effectiveness of the proposed method, which are either a typical MCPS or some important constituent protocols of MCPSs.

### 4.1. Dynamic Power Management

This case is the power dynamics management of the IBM disk drive [38,39]. It is a typical MCPS, and can be used for various areas, e.g., multi-robot collaboration. Its purpose is to minimize the power consumption while minimizing the impact on performance. It is modeled as a DTMC in PAT with approximately 140,000 states and transitions. We are interested in the event that is 100 or more requests getting lost within 400 milliseconds. The time consumption of transition in DTMC is 0.5 milliseconds, and the step number of property is 800. Therefore, the state formula corresponding to this event is $\left(true\;U{\;}^{\leq 800}lost=100\right)$. The probability calculated by PAT is 0.014725297, if the time-bound property of $P_{<0.014725297}\left(true\;U^{\leq 800}\;lost=100\right)$ is violated, which is a time-bound property of DTMC. We generate the counterexample for it by HGA, GA, XZ, XUZ, and Eppstein algorithms.

**Figure 6:**
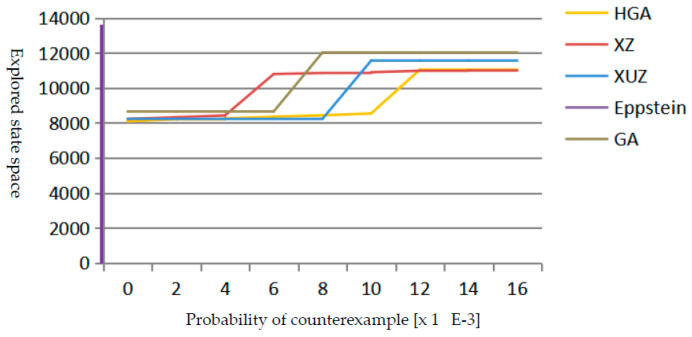
Counterexample probability of power dynamics management.

As shown in Figure 6, it is obvious that the Eppstein algorithm is almost parallel to the y-axis for a counterexample probability close to zero. HGA, XZ, and XUZ can successfully provide counterexamples for all possible upper bounds with a reasonable computational effort. HGA finds the first increment of the counterexample after exploring a smaller fraction of the state space than XZ and XUZ. The Probability value of the first increment which is generated by HGA is greater than 0.011, and the reason for this is that we use a diagnostic subgraph to represent counterexample. After exploring about 2000 states and transitions, all algorithms can implement the second increment of the counterexample, and generate a counterexample after finding the second increment, because the total probability value exceeds the given probability. In the process of finding a counterexample, HGA explores fewer states and transitions than XZ and XUZ algorithms, except for the range between 0.006 and 0.011. HGA reaches the probability bound within about 3 s and consumes 250 KB memory. Meanwhile, GA, XZ, and XUZ algorithms reach the probability bound within about 3 s and 300 KB. GA generates a counterexample exploring more states and transitions, because it uses directed coding methods to generate many invalid paths and crossover operators used by GA cannot generate better offspring. 

### 4.2. Synchronous Leader Election Protocol

This case is the synchronous leader election protocol [40,41]. It selects a unique leader node from *N* identical network nodes of the MCPSs. At each round, every node chooses a random number from {0,…,K} as its ID. A node with the maximum unique ID will be elected as leader; otherwise, a new round begins between these nodes. The protocol system is modeled in PAT as a DTMC with 12,400 states and 16,495 transitions. Here, we set *N* and *K* as 4 and 8, respectively. This process will not stop until the leader is elected. The probability of the formula (F<=(L∗(N+1) “elected”)) is 0.95703125 by PAT, where “elected” indicates that a node has been selected as the leader, and *L* is set to be 1. We generate a counterexample of property $P_{\leq 0.95703125}(F<=\left(L*\left(N+1\right)\;“elected”\right))$ by HGA, GA, XZ, XUZ, and Eppstein algorithms.

**Figure 7:**
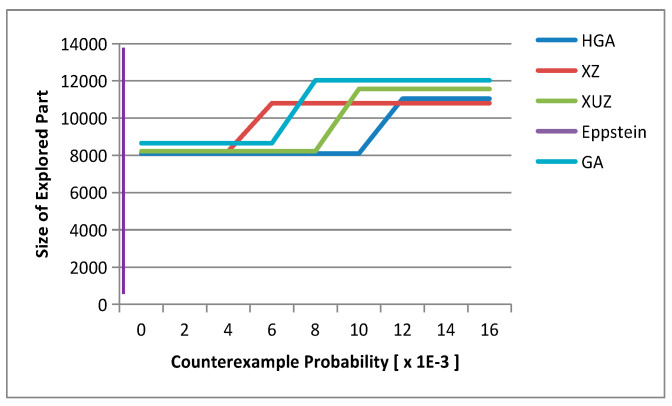
Counterexample probability of leader election.

As shown in Figure 7, we can see that the Eppstein and XUZ algorithms grow almost parallel to the y-axis, so the probability of the counterexample is zero. HGA and XZ can generate a counterexample for probability upper bound by reasonable computational effort. The HGA finds the first increment after exploring about half of the entire state space. However, the probability of the first increment of counterexample is less than 0.5. After exploring all the states and transitions, the HGA and XZ algorithms can find the second increment. HGA explores fewer states and transitions than XZ; and HGA finds the first increment with consumption of about 150 s and 360 KB memory. The GA algorithm consumes too much memory to find a counterexample, the reason being that direct coding produces more invalid paths. 

### 4.3. Zeroconf Protocol

This case is a Zeroconf protocol [42]. It offers a distributed “plug-and-play” solution, in which the address configuration is managed by an individual MCPSs device when it is connected to the network. Each station has a single-message buffer and is cyclically attended by the server. The buffer could store the messages that it wants to send; in such cases, messages are not relevant after reconfiguring, and thus keeping these messages can slow down the network and make hosts reconfigure when they do not need to. We only consider version networks where the host does not do anything (No Reset) on these messages. 

It is modeled as an MDP in PAT. The number of abstract hosts is denoted by *N*, the number of probes to send is denoted by *K*, and the probability of message loss is denoted by a loss. In this experiment, *N* and loss are set to 1000 and 0.1, respectively. *K* is set to 2 or 4. We are interested in the probability that a host picks an IP address already in use. In Table 1, “Time” and “Memory” represent the run time (in seconds) and memory (in KB). We compare Eppstein, XUZ, GA, and HGA on the two k-value variants of the model, as shown in Table 2 and Table 3. When the searched state space is very big, Eppstein and XUZ algorithms cannot get a counterexample. They are recorded as “Failed”, which denotes out of memory. The time consumption of HGA is less than Eppstein, XUZ, and GA, at the expense of very little memory, especially when the searched state and transitions are very large. 

### 4.4. Bounded Retransmission Protocol

BRP (bounded retransmission protocol) is a variant of the alternating bit protocol [43] for the compositional MCPSs. It sends a file in several chunks, but allows only a bounded number of retransmissions of each chunk. It is modeled as an MDP in PAT, where *N* represents the number of blocks, and MAX represents the maximum number of retransmits allowed for each block. In the experiment, MAX is set to 5 and the size of state space is scaled by *N*. It is modeled as an MDP in PAT; we are interested in the probability that the sender does not report a successful transmission. Model information is shown in Table 4, and experimental results are shown in Table 5 and Table 6, the fields of which have the same meaning as Table 1, Table 2 and Table 3, respectively.

The non-deterministic analysis of MDP results in 137,313 transitions at N = 640 and 685,153 transitions at N = 3200. As can be seen from Table 4, the transitions in DTMC are 98% of that in MDP, which indicates that the MDP model is low uncertainty. Table 5 and Table 6 list very low probabilities because these methods fail to provide counterexamples for large probability boundaries in real time. This is caused by the very low probability of a single diagnostic path being carried in this model, which means that many diagnostic paths must be found and processed to increase the probability of counterexamples. The run time and memory consumption of HGA performs best, especially for large probability bounds. HGA has a greater advantage in memory consumption because more efficient paths are found with a heuristic, and for large probability boundaries, the number of diagnostic paths found is very large, so there is a great advantage in storing them in an AND/OR tree. 

### 4.5. Analysis

Compared with the existing works, we can see that HGA can generate a counterexample effectively for all 4 cases, even if the Eppstein algorithm, XUZ algorithm, and Genetic algorithm cannot get the counterexample for large state space of MCPSs, which are noted as “failed” in the corresponding tables. The bigger the state space of MCPSs, the more the effectiveness of our method is apparent, compared with the related works. This is rooted in the advantages of HGA: firstly, under the action of the genetic operator, HGA has a strong search ability, which can find the global optimal solution of the counterexample with a large probability; secondly, inherent in the parallelism of HGA, it can effectively deal with the large state space of the MCPSs model MDP. Compared with GA, HGA also performs better in counterexample generation for MDP. It is because that we adopt the indirect path coding in HGA to extend the search range of state space, and the heuristic crossover operator to generate more effective diagnostic paths.

## 5. Conclusions

We propose an HGA-based counterexample generation for PCTL probabilistic model checking MCPSs with nondeterministic and discrete-time stochastic behaviors, i.e., MDP. As far as we know, it is the first counterexample generation method that employs the Genetic algorithm to search the diagnostic subgraph of MDP. We encode paths into chromosomes to generate more efficient paths through priority-based indirect coding. The heuristic crossover operator guarantees that a number of populations are excellent individuals, which helps to improve the average value of the population and speed up the convergence. We use the diagnostic subgraph to represent the counterexample, which is more compact. Experimental results show that the time and memory consumption are less than existing works. In the future, we will continue to optimize HGA to generate counterexamples for MCPSs against requirements in PCTL* which is a super set of PCTL. At the same time, we would like to extend HGA to generate counterexamples for MCPSs models with continuous-time or hybrid-time stochastic behaviors, such as CTMDPs (Continuous-time Markov Decision Processes), PHA (Probabilistic Hybrid Automata), against CSL (Continuous-time Stochastic Logic) stochastic communication logics [44,45].

## Figures and Tables

**Table 1:** Model information of MDP in PAT.

K	2	4
States	77,279	276,781
Transitions	180,442	640,721
Time	24.868	77.430
Memory	3584.0	11,878.4

**Table 2:** Run time of counterexample generation.

K	Probability	Eppstein	XUZ	GA	HGA
2	0.01	19.076	17.161	16.105	15.176
	0.04	169.06	165.090	156.223	105.103
	0.08	909.082	801.214	601.31	520.282
	0.1	2198.009	1996.071	1077.56	809.072
4	0.01	93.040	105.943	37.09	29.235
	0.04	786.047	518.177	509.43	435.034
	0.08	5963.085	4163.076	2607.38	1998.089
	0.1	Failed	Failed	5901.92	4076.92

**Table 3:** Memory consumption of counterexample generation.

K	Probability	Eppstein	XUZ	GA	HGA
2	0.01	797.9	702.8	699.4	690.2
	0.04	3798.4	3301.7	2863.5	2831.3
	0.08	7802.2	7032.9	5051.2	4960.3
	0.1	18,915.6	16,970.3	10,967.9	9818.9
4	0.01	7849.2	7649.3	7734.6	5782.34
	0.04	37,851.7	37,091.8	15,736.6	9825.4
	0.08	87,869.7	85,981.8	36,751.5	17,838.3
	0.1	Failed	Failed	99,302.2	48,804.2

**Table 4:** Model information of MDP in PAT.

N	640	3200
States	103,962	518,682
Transitions	139,888	697,968
Time	347.055	5023.233
Memory	4403.2	20,992.0

**Table 5:** Run time of counterexample generation.

N	Probability	Eppstein	XUZ	GA	HGA
640	0.01	132.721	120.835	111.443	109.234
	0.04	633.893	626.542	496.856	409.764
	0.08	5328.984	4996.132	1975.362	1153.762
	0.10	Failed	Failed	4206.716	2043.571
3200	0.005	1402.754	1603.239	1085.234	987.365
	0.008	5405.326	5564.271	2354.331	1801.259
	0.016	Failed	Failed	5979.845	2969.267
	0.02	Failed	Failed	Failed	8597.813

**Table 6:** Memory consumption of counterexample generation.

N	Probability	Eppstein	XUZ	GA	HGA
640	0.01	13,009.5	13,447.4	12,950.1	12,908.3
	0.04	23,768.1	24,647.8	23,840.4	21,987.2
	0.08	514,153.4	528,356.3	49,453.2	77,623.2
	0.10	Failed	Failed	Failed	893,615.8
3200	0.005	549,063.4	43,252.3	30,432.2	29,987.5
	0.008	793,109.2	797,602.6	618,023.1	50,568.2
	0.016	Failed	Failed	896,581.1	93,298.4
	0.02	Failed	Failed	Failed	897,536.8

## Abbreviations

**Full Name**	**Abbreviation**
Micro-scale Cyber-Physical Systems	MCPSs
Micro-scale Cyber-Physical System	MCPS
Discrete-time Markov Chains	DTMCs
Markov Decision Processes	MDPs
Probabilistic tic Timed Automata	PTA
Probabilistic Computation Tree Logic	PCTL
Liner Temporal logic	LTL
Probabilistic Timed Computation Tree Logic	PTCTL
Bounded Model Checking	BMC
Best-Frist	BF
Genetic Algorithm	GA
Heuristic Genetic Algorithm	HGA
Bounded Retransmission Protocol	BRP
Continuous-time Markov Decision Processes	CTMDPs
Probabilistic Hybrid Automata	PHA
Continuous-time Stochastic Logic	CSL
